# Benchmarking Pre-National Action Plan Systemic Antimicrobial Consumption in Tanzania, 1998-2005: A Historical Longitudinal Defined Daily Dose Analysis

**DOI:** 10.7759/cureus.98729

**Published:** 2025-12-08

**Authors:** Raphael Z Sangeda, Auleria W Kadinde, Akida Khea, Adam M Fimbo

**Affiliations:** 1 Department of Pharmaceutical Microbiology, Muhimbili University of Health and Allied Sciences, Dar es Salaam, TZA; 2 Department of Regulation and Prequalification, World Health Organization, Copenhagen, DNK; 3 Medicines Control, Tanzania Medicines and Medical Devices Authority, Dodoma, TZA

**Keywords:** antibiotics, antimicrobial resistance, antimicrobial stewardship, defined daily dose, systemic antimicrobials, tfda, tmda, utilization

## Abstract

Background

Antimicrobial consumption (AMC) data in Tanzania prior to the 2017 National Action Plan on Antimicrobial Resistance (NAP-AMR) are scarce, limiting our understanding of historical usage patterns.

Objective

To establish a national baseline of systemic AMC in Tanzania from 1998 to 2005 using the World Health Organization's Defined Daily Dose (DDD) methodology.

Methods

A retrospective longitudinal analysis of antimicrobial import records from the Tanzania Medicines and Medical Devices Authority was conducted, expressing consumption as DDD per 1,000 inhabitants per day (DID).

Results

Between 1998 and 2005, annual AMC ranged from 0.45 to 13.32 DID, predominantly antibacterials (82.6%), with cumulative consumption of 51.9 DID. Oral formulations and Access category antibiotics dominated usage.

Conclusion

These historical AMC estimates provide a valuable benchmark for contextualizing and informing future antimicrobial stewardship efforts in Tanzania.

## Introduction

The prudent use of antimicrobials is central to limiting the emergence and spread of antimicrobial resistance (AMR) [1]. In Tanzania, understanding historical utilization patterns is important because these data inform antimicrobial stewardship (AMS) programme design and contextualize subsequent national antimicrobial consumption (AMC) datasets. Several national analyses have characterized antimicrobial utilization after 2010 using importation data [2-5], whereas other studies have examined facility-level utilization patterns [6], inappropriate dispensing practices [7], and geographical heterogeneity in antimicrobial consumption [2]. Implementation research has also documented early progress in AMS following the launch of Tanzania's National Action Plan (NAP) on AMR in 2017 [3,4]. However, little is known about the use of systemic antimicrobials, including antibacterials, antivirals, antifungals, and anti-tuberculosis agents, in Tanzania prior to this policy period. To address this gap, we present a national benchmark for antimicrobial consumption between 1998 and 2005, before intensified AMR containment efforts. Establishing this early baseline enables a more accurate interpretation of subsequent national datasets and helps demonstrate how antimicrobial use changed as stewardship frameworks matured. It also provides a reference point for comparing pre- and post-2010 data generated by the national regulatory authority and contextualizing the shifts associated with antimicrobial stewardship initiatives.

This study aimed to generate a historical national baseline for systemic antimicrobial consumption in Tanzania for the period 1998-2005. Specifically, this study quantified the import-based consumption of antibacterials, antivirals, antifungals, and anti-tuberculosis agents using the World Health Organization (WHO) Anatomical Therapeutic Chemical (ATC)/Defined Daily Dose (DDD) methodology and expressed utilization as DDD per 1,000 inhabitants per day (DID) [8].

## Materials and methods

Study design and period

This was a retrospective longitudinal study describing the national utilization of all systemic antimicrobials for human use in Tanzania between 1998 and 2005. Topical preparations and nonhuman products were excluded from the study. Antibacterials were additionally classified using the WHO AWaRe framework; however, the AWaRe categorization was not applied to antivirals, antifungals, or anti-tuberculosis agents.

Regulatory context

Between 1998 and 2005, pharmaceutical import records in Tanzania were generated under regulatory arrangements that predated the current authority. Prior to 2003, the approval of pharmaceutical imports and product registration was overseen by the Ministry of Health through the Pharmacy Board established under the Pharmaceuticals and Poisons Act of 1978. In July 2003, these functions were transferred to the newly operational Tanzania Food and Drugs Authority (TFDA) under the Tanzania Food, Drugs and Cosmetics Act (Cap 219), which assumed responsibility for regulating medicines, medical devices, foods, and cosmetics. In 2019, TFDA was renamed the Tanzania Medicines and Medical Devices Authority (TMDA) following legislative amendments that reassigned food and cosmetics regulations to the Tanzania Bureau of Standards (TBS) [5]. These institutional transitions have influenced the evolution of import-permit information systems over time; however, the present analysis applies a consistent WHO ATC/DDD methodology across all study years.

Data source

Annual importation records for systemic antimicrobial products were retrieved from the national regulatory authority, the TFDA, from 1998 to 2005, now the TMDA. During the study period, import permit records were captured using locally deployed Microsoft Access (Microsoft Corp., Redmond, WA, USA)-based regulatory information systems for registered and imported products (RegSoft and InspectSoft). These platforms stored structured import-permit data before later migrating into SQL-based regulatory databases, which were subsequently integrated into TMDA's modern Regulatory Information Management System (RIMS) [6]. Products were classified into four antimicrobial groups (antibacterial, antiviral, antifungal, and anti-tuberculosis agents). Imported quantities of systemic antimicrobials were used as a proxy for national utilization. Complete permit records for all study years were available, and no imputation of missing data was performed.

Antibiotic classification

All systemic antimicrobial products were mapped to the WHO ATC classification system to enable consistent year-to-year comparisons across the study period [8]. Antibacterials were further categorized using the WHO AWaRe framework (Access, Watch, Reserve) [7]. The AWaRe stratification was not applied to antivirals, antifungals, or anti-tuberculosis agents.

Consumption quantification and population denominators

The AMC was standardized using the WHO's DDD methodology. Annual totals were expressed as DID [9]. Annual population estimates were obtained from the National Bureau of Statistics (NBS) of Tanzania.

The population figures used as denominators for each calendar year in this analysis were derived from intercensal and mid-year population projections anchored to the official 2002 Population and Housing Census published by the NBS of Tanzania. The 2002 census population (34,443,603) was obtained directly from the NBS report Population Distribution by Enumeration Area [10]. Annual population values for the surrounding years (1999-2005) were generated using NBS-consistent demographic growth assumptions applied between census rounds to produce a continuous, internally consistent projection series suitable for longitudinal analyses.

Data analysis

Summary values were produced for each calendar year and compared descriptively to assess absolute changes and year-to-year variability in antimicrobial consumption. Patterns across antimicrobial classes (antibacterial, antiviral, antifungal, and anti-tuberculosis agents) were reviewed to illustrate the heterogeneity of utilization within the pre-National Action Plan period. All analyses were descriptive, and no inferential statistical modelling was performed. Descriptive summaries, DID calculations, and yearly trend visualizations were generated using Microsoft Power BI (Microsoft Corp., Redmond, WA, USA).

Ethical considerations

Ethical approval for research involving administrative TMDA data was granted by the Muhimbili University of Health and Allied Sciences (MUHAS) Institutional Review Board (Reference: DA.25/111/01B/182). Permission to access and analyze historical import permit records was granted by the Tanzania Medicines and Medical Devices Authority (TMDA). The dataset contained no patient-level or personally identifiable information, and only aggregated administrative data were used. Informed consent was not required for this study.

## Results

**Figure 1:**
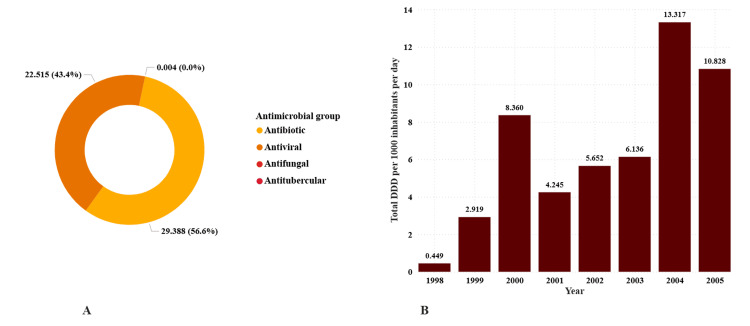
Systemic antimicrobial consumption by antimicrobial group (Panel A) and by year (Panel B) in Tanzania from 1998 to 2005, expressed in defined daily dose per 1,000 inhabitants per day (DID). Total systemic antimicrobial consumption by antimicrobial group (Panel A) is expressed in DID and percentage, while annual systemic antimicrobial consumption (Panel B) is in DID only.

Between 1998 and 2005, 8,010 systemic antimicrobial import entries were identified after exclusion, comprising antibacterials (n = 6,619; 82.6%), antivirals (n = 892; 11.1%), antifungals (n = 492; 6.1%), and anti-tuberculosis agents (n = 7; 0.1%). The cumulative consumption across all years was 51.9063 DID, with contributions from antibacterials at 29.39 (≈56.6%), antivirals at 22.51 (≈43.4%), antifungals at 0.0035 (<0.01%), and anti-tuberculosis agents at 0.0003 (<0.01%). Annual total antimicrobial consumption rose from 0.45 DID in 1998 to peaks of 8.36 DID in 2000 and 13.32 DID in 2004, before decreasing to 10.83 DID in 2005 (Figure 1).

**Figure 2:**
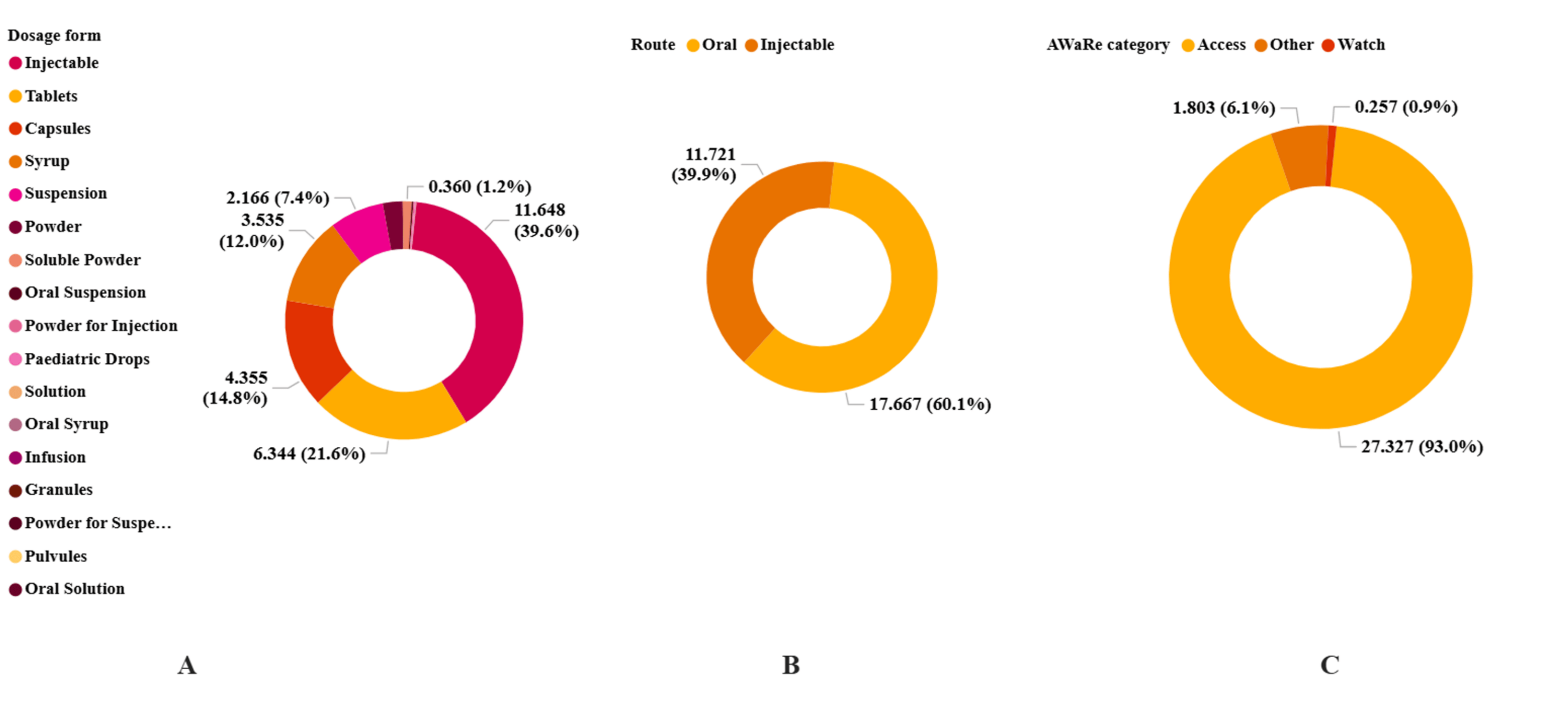
Distribution of antibacterial consumption expressed as defined daily dose per 1,000 inhabitants per day (DID) by dosage form (Panel A), route of administration (Panel B) and Access, Watch and Reserve (AWaRe) category (Panel C) in Tanzania from 1998 to 2005. Presenting DID and the percentage contribution of each antibacterial dosage form (Panel A), route of antimicrobial administration (Panel B) and by AWaRe category (Panel C). No Reserve group antibacterials were identified during the study period.

When utilization was stratified by dosage form, oral liquid products (suspension and syrup) were dominant: suspension contributed 0.212 DID (35.97%) and syrup contributed 0.146 DID (24.80%), injectables contributed 0.087 DID (14.79%), and capsules and tablets contributed 0.085 DID (14.50%) and 0.057 DID (9.71%), respectively (Figure 2, Panel A). Oral formulations accounted for 0.501 DID (84.99%), whereas injectables contributed 0.088 DID (15.01%) (Figure 2, Panel B). Under the AWaRe framework, Access to antibiotics comprised 0.563 DID (95.56%), with small contributions from other antibiotics (0.026 DID, 4.36%) and Watch antibiotics (0.0004 DID, 0.07%) (Figure 2, Panel C). No Reserve antibiotics were identified in the dataset.

**Figure 3:**
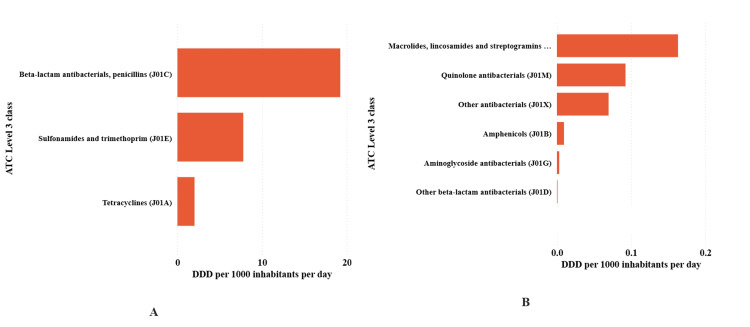
Antibacterial consumption categorized using the Anatomical Therapeutic Chemical Level 3 classification expressed as defined daily dose per 1,000 inhabitants per day (DID) in Tanzania from 1998 to 2005. Panel A shows Level 3 classes contributing ≥2 DID, including penicillins, sulfonamides with trimethoprim, and tetracyclines. Panel B shows the remaining Level 3 classes contributing ≤2 DID.

At ATC level 3, beta-lactam penicillins (J01C) accounted for 19.24 DID (65.5%), followed by sulfonamides and trimethoprim (J01E) at 7.78 (26.5%) and tetracyclines (J01A) at 2.03 (6.9%). All other ATC Level 3 antibacterials each contributed ≤1% in total, including macrolides/lincosamides/streptogramins (J01F) 0.16 (0.6%), quinolones (J01M) 0.09 (0.3%), other antibacterials (J01X) 0.07 (0.2%), amphenicols (J01B) 0.01 (<0.1%), aminoglycosides (J01G) 0.003 (<0.01%) and other beta-lactams (J01D) 0.001 (<0.01%) (Figure 3).

**Figure 4:**
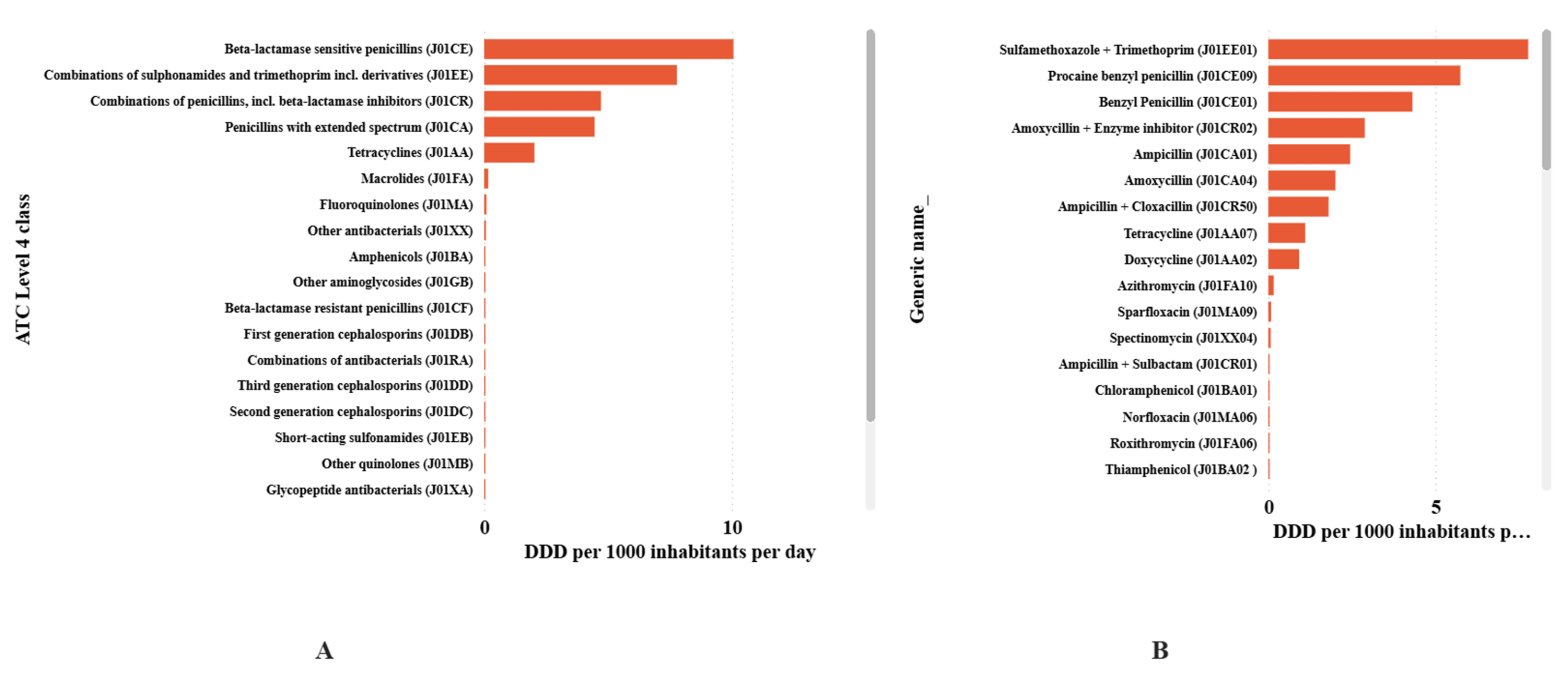
Antibacterial consumption categorized using the Anatomical Therapeutic Chemical Level 4 (Panel A) and Level 5 (Panel B) classifications expressed as defined daily dose per 1,000 inhabitants per day in Tanzania from 1998 to 2005.

The patterns of antibacterial utilization, according to the ATC classification, are presented in Figure 4. At ATC Level 4 (Panel A), the largest cumulative DID contributions were from beta-lactamase-sensitive penicillins and sulphonamide/trimethoprim combinations, followed by extended-spectrum penicillins and penicillins combined with beta-lactamase inhibitors. At ATC Level 5 (Panel B), the highest DID values were observed for sulfamethoxazole + trimethoprim, procaine benzyl penicillin, and benzyl penicillin. Appendix 1 and Appendix 2 list the complete ATC Level 4 and Level 5 antibacterial products, along with their associated DID values.

**Figure 5:**
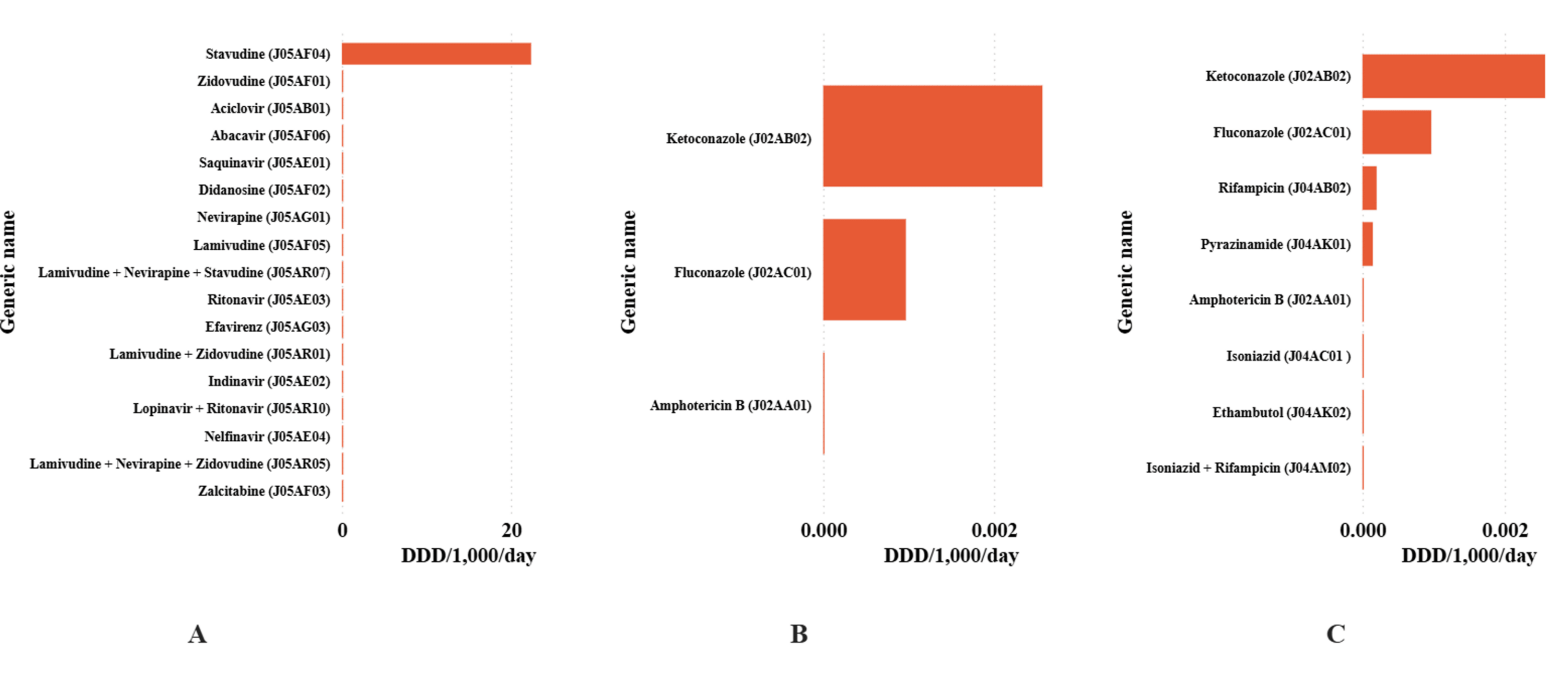
Antiviral (Panel A), antifungal (Panel B) and anti-tuberculosis (Panel C) substances categorized using the Anatomical Therapeutic Chemical Level 5 classification expressed as defined daily dose per 1,000 inhabitants per day in Tanzania from 1998 to 2005.

The antiviral, antifungal, and anti-tuberculosis substances identified in the dataset for 1998-2005 at ATC Level 5 are shown in Figure 5. Among antivirals (Panel A), stavudine accounted for 22.386584 DID (99.4% of total antiviral DID), zidovudine for 0.093740 (0.4%), and acyclovir for 0.024338 (0.1%). Among the systemic antifungal agents (Panel B), ketoconazole (0.002568 DID) and fluconazole (0.000968 DID). Panel C lists anti-tuberculosis substances, along with their associated DID values.

**Figure 6:**
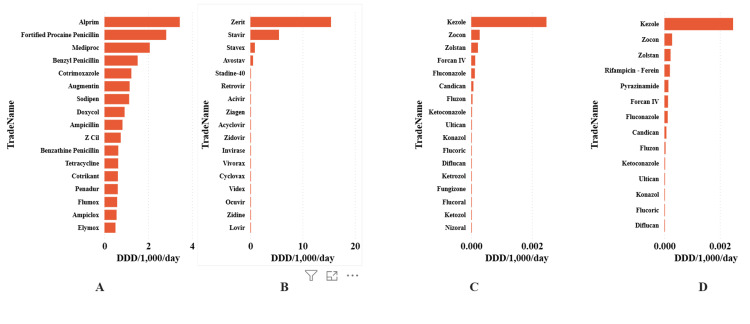
Top trade name-specific consumption of antibacterial (Panel A), antiviral (Panel B), antifungal (Panel C) and anti-tuberculosis (Panel D) products expressed as defined daily dose per 1,000 inhabitants per day in Tanzania from 1998 to 2005. Showing only up to the top 17 products.

At ATC Level 5, a limited number of trade names accounted for the majority of the measurable DID contributions. Figure 6 presents the highest-contributing products per antimicrobial category, with Panels A-D showing the top observed trade names for antibacterials, antivirals, antifungals, and anti-tuberculosis agents. The values are expressed as DID. The top 17 trade names collectively accounted for 66.3% of antibacterial DID, 100.0% of antiviral DID, 98.3% of antifungal DID, and 97.9% of anti-tuberculosis DID.

## Discussion

This analysis provides a historical national reference point for systemic antimicrobial imports into Tanzania prior to intensified AMR containment efforts. Previous national TMDA import-based analyses, using the same ATC/DDD methodology, have quantified AMC from 2010 to 2022 [6,11-14]. Facility-level surveys have also described utilization patterns at national referral hospitals [15], regional referral hospitals [16,17], and within specialized inpatient settings [18]. Therefore, the present 1998-2005 estimates extend the national AMC timeline backward by more than a decade, providing an early baseline that can be compared with the post-2010 TMDA import-based series using the same ATC/DDD methodology. However, differences in regulatory structures and data capture systems across these periods warrant cautious interpretation.

Compared with later national datasets (2010-2016, 2010-2017, and 2020-2022), the 1998-2005 period was characterized by lower overall DID values and a narrower range of antimicrobial agents. This profile reflects the market structure, product availability, and treatment policies that preceded the rollout of intensified AMR containment efforts after 2017. Later analyses reported higher DID estimates and greater representation of later-generation agents, which is consistent with market evolution, shifts in treatment guidelines, and expanded AMS activities [6,11-13].

Non-antibiotic consumption during 1998-2005 was extremely low, dominated by stavudine among antivirals and by ketoconazole and fluconazole among antifungals, with anti-tuberculosis agents contributing minimally to the overall DID. These patterns reflect the pre-ART scale-up era and the limited availability of systemic antifungal therapy. Later, national data indicated substantial increases in systemic antiviral and antifungal consumption from 2010 to 2017 [11] and a partial omission of these classes in the 2017-2019 AMC analysis [14]. In contrast, the 2020-2022 AMC series reported further overall increases, albeit with limited class-specific details [13]. The 1998-2005 estimates, therefore, represent the earliest national ATC/DDD baseline for non-antibacterial agents, helping to contextualize the expansions observed in subsequent years.

When compared with global antibiotic consumption trends, the molecular profile of Tanzania's imports from 1998 to 2005 aligns with a pre-transition antimicrobial market dominated by older narrow-spectrum agents. Globally, the period from 2000 to 2010, and later extended to 2018, was characterized by the increasing use of later-generation classes, including carbapenems, polymyxins, third-generation cephalosporins, macrolides, and fluoroquinolones [19,20]. In contrast, the Tanzanian market remained heavily shaped by co-trimoxazole (26.5% of the total DID), legacy injectable penicillins (procaine benzylpenicillin and benzylpenicillin, combined, ~35%), and broad-spectrum penicillins, such as ampicillin and amoxicillin. Macrolides, fluoroquinolones, cephalosporins, and carbapenems contributed minimally to the national DID. This pattern reflects both the regulatory and procurement contexts of the period as well as the slower introduction of later-generation agents compared to trends in low- and middle-income countries globally.

The dominance of a limited number of antibiotics was notable. The top 17 trade names accounted for 66.3% of antibacterial DID, 100.0% of antiviral DID, 98.3% of antifungal DID, and 97.9% of anti-tuberculosis DID. This concentration suggests a market dominated by legacy penicillin formulations and co-trimoxazole combinations during the 1998-2005 period, with relatively few later-generation or Reserve-category agents entering the market.

Limitations

This analysis used national importation records as a proxy for utilization. Import-based estimates do not measure actual AMC or dispensing volumes and cannot distinguish between hospital and retail sector distributions. Data were captured using locally deployed Microsoft Access-based regulatory information systems, and the completeness of the records within this period may have varied. Product-level matching at the formulation and pack-strength levels was dependent on import-permit coding, and the post-import stock movement was not traceable. Granular stratification by region, facility type, or public vs. private sector was not available.

## Conclusions

This study provides the earliest national ATC/DDD-based estimates of systemic antimicrobial imports in Tanzania, establishing a foundational baseline for the pre-NAP-AMR era (1998-2005). Antimicrobial consumption during this period was dominated by Access-category beta-lactam penicillins and co-trimoxazole, with minimal uptake of later-generation agents. When interpreted alongside post-2010 TMDA antimicrobial consumption datasets, these findings highlight the structural, regulatory, and market transitions that shaped antimicrobial use prior to the expansion of national AMS activities.

These historical estimates serve as an important benchmark for evaluating long-term consumption trends, guiding AMS programme assessments, and informing AMR containment policy in Tanzania. As AMS efforts mature, integrating import-based metrics with dispensing records and facility-level utilization data will help generate a more comprehensive understanding of national antimicrobial use and support more targeted interventions. Taken together, these findings provide essential public health context to strengthen evidence-based decision-making and national AMS initiatives.

## Appendices

Appendix 1 

**Table 1:** Antibacterial consumption categorized using the Anatomical Therapeutic Chemical (ATC) Level 4 classification in Tanzania, 1998–2005. Antibacterial groups are presented using Level 4 ATC codes in brackets and are sorted by descending contribution to consumption. DID = defined daily dose per 1,000 inhabitants per day; % = percentage share of total antibacterial consumption (denominator = 29.387795 DID).

ATC Level 4 class	DID	%
Beta-lactamase-sensitive penicillins (J01CE)	10.065766	34.2
Combinations of sulphonamides and trimethoprim, including derivatives (J01EE)	7.783053	26.5
Combinations of penicillins incl. beta-lactamase inhibitors (J01CR)	4.714140	16.0
Penicillins with extended spectrum (J01CA)	4.459220	15.2
Tetracyclines (J01AA)	2.026624	6.9
Macrolides (J01FA)	0.163165	0.6
Fluoroquinolones (J01MA)	0.092392	0.3
Other antibacterials (J01XX)	0.069610	0.2
Amphenicols (J01BA)	0.009498	0.0
Other aminoglycosides (J01GB)	0.003096	0.0
Beta-lactamase-resistant penicillins (J01CF)	0.000383	0.0
First-generation cephalosporins (J01DB)	0.000355	0.0
Combinations of antibacterials (J01RA)	0.000190	0.0
Third-generation cephalosporins (J01DD)	0.000145	0.0
Second-generation cephalosporins (J01DC)	0.000069	0.0
Short-acting sulfonamides (J01EB)	0.000064	0.0
Other quinolones (J01MB)	0.000010	0.0
Glycopeptide antibacterials (J01XA)	0.000009	0.0
Lincosamides (J01FF)	0.000003	0.0
Fourth-generation cephalosporins (J01DE)	0.000001	0.0
Carbapenems (J01DH)	0.000000	0.0
Streptomycins (J01GA)	0.000000	0.0

Appendix 2

**Table 2:** Antibacterial consumption categorized using the Anatomical Therapeutic Chemical (ATC) Level 5 classification in Tanzania, 1998–2005. Antibacterial substances are presented using ATC Level 5 with codes in brackets and are sorted by descending contribution to consumption. DID = defined daily dose per 1,000 inhabitants per day; % = percentage share of total antibacterial consumption (denominator = 29.387795 DID); CUM % = cumulative percentage used to identify drug utilization (DU) 50 and DU 90 thresholds.

Generic product (ATC L5)	DID	%	CUM %
Sulfamethoxazole + Trimethoprim (J01EE01)	7.783049	26.5	26.5
Procaine benzyl penicillin (J01CE09)	5.749908	19.6	46.0
Benzyl Penicillin (J01CE01)	4.315676	14.7	60.7
Amoxycillin + Enzyme inhibitor (J01CR02)	2.886470	9.8	70.6
Ampicillin (J01CA01)	2.448990	8.3	78.9
Amoxycillin (J01CA04)	2.010230	6.8	85.7
Ampicillin + Cloxacillin (J01CR50)	1.803319	6.1	91.9
Tetracycline (J01AA07)	1.104642	3.8	95.6
Doxycycline (J01AA02)	0.921976	3.1	98.8
Azithromycin (J01FA10)	0.158205	0.5	99.3
Sparfloxacin (J01MA09)	0.083324	0.3	99.6
Spectinomycin (J01XX04)	0.069610	0.2	99.8
Ampicillin + Sulbactam (J01CR01)	0.023915	0.1	99.9
Chloramphenicol (J01BA01)	0.007407	0.0	99.9
Norfloxacin (J01MA06)	0.006963	0.0	100.0
Roxithromycin (J01FA06)	0.003856	0.0	100.0
Thiamphenicol (J01BA02)	0.002091	0.0	100.0
Ciprofloxacin (J01MA02)	0.001793	0.0	100.0
Amikacin (J01GB06)	0.001753	0.0	100.0
Tobramycin (J01GB01)	0.001223	0.0	100.0
Erythromycin (J01FA01)	0.001064	0.0	100.0
Sultamicillin (J01CR04)	0.000436	0.0	100.0
Cloxacillin (J01CF02)	0.000377	0.0	100.0
Pefloxacin (J01MA03)	0.000215	0.0	100.0
Cefadroxil (J01DB05)	0.000197	0.0	100.0
Norfloxacin + Tinidazole (J01RA13)	0.000190	0.0	100.0
Phenoxymethyl Penicillin (J01CE02)	0.000183	0.0	100.0
Cefalexin (J01DB01)	0.000158	0.0	100.0
Ceftriaxone (J01DD04)	0.000138	0.0	100.0
Gentamycin (J01GB03)	0.000121	0.0	100.0
Ofloxacin (J01MA01)	0.000072	0.0	100.0
Sulfadimidine (J01EB03)	0.000064	0.0	100.0
Cefuroxime (J01DC02)	0.000044	0.0	100.0
Cefaclor (J01DC04)	0.000023	0.0	100.0
Clarithromycin (J01FA09)	0.000020	0.0	100.0
Clarithromycin + Lansoprazole + Tinidazole (J01FA09)	0.000020	0.0	100.0
Lomefloxacin (J01MA07)	0.000011	0.0	100.0
Nalidixic Acid (J01MB02)	0.000010	0.0	100.0
Levofloxacin (J01MA13)	0.000010	0.0	100.0
Vancomycin (J01XA01)	0.000009	0.0	100.0
Flucloxacillin (J01CF05)	0.000006	0.0	100.0
Oxytetracycline (J01AA06)	0.000004	0.0	100.0
Cefotaxime (J01DD01)	0.000004	0.0	100.0
Sulfadiazine + Trimethoprim (J01EE02)	0.000004	0.0	100.0
Gatifloxacin (J01MA16)	0.000003	0.0	100.0
Clindamycin (J01FF01)	0.000003	0.0	100.0
Moxifloxacin (J01MA15)	0.000002	0.0	100.0
Cefprozil (J01DC10)	0.000002	0.0	100.0
Minocycline (J01AA08)	0.000002	0.0	100.0
Ceftazidime (J01DD02)	0.000001	0.0	100.0
Cefepime (J01DE01)	0.000001	0.0	100.0
Cefixime (J01DD08)	0.000001	0.0	100.0
Meropenem (J01DH02)	0.000000	0.0	100.0
Streptomycin (J01GA01)	0.000000	0.0	100.0
Clarithromycin + Lansoprazole + Tinidazole (A02BD09)	0.000000	0.0	100.0
